# Direct measurement of non-thermal microwave effects on bacterial growth and redox dynamics using a novel high-throughput waveguide applicator

**DOI:** 10.1098/rsta.2024.0073

**Published:** 2025-05-22

**Authors:** Angharad Miles, Adrian Porch, Heungjae Choi, Steve Cripps, Helen Brown, Catrin Williams

**Affiliations:** ^1^ School of Engineering, Cardiff University, Cardiff, UK; ^2^ School of Biosciences, Cardiff University, Cardiff, UK

**Keywords:** bacteria, pulsed, electric field

## Abstract

A high-throughput microwave applicator has been designed and characterized to investigate microwave interactions with biological systems. When operated in the TE_10_ mode, this rectangular waveguide enabled simultaneous exposure of 96 biological samples to a quantifiable electric field (*E* field) at 2.45 GHz. Optimized electric probe transitions efficiently couple power (up to 50 W) into and out of the waveguide, achieving a voltage transmission coefficient (S_21_) near unity (0 dB) and a voltage reflection coefficient (S_11_) below 0.01 ( less than −20 dB). The growth dynamics of *Staphylococcus aureus* bacteria were analysed after non-thermal, microsecond-pulsed microwave exposure at 25 W r.m.s. of microwave power for 24 h. Post-exposure, *S. aureus* exhibited significantly higher optical density measurements and growth rates than thermal controls. Fluorescent probes directed towards key redox indicators revealed that microwave exposure altered the cellular redox state. This study provides new insights into the non-thermal effects of pulsed 2.45 GHz microwaves on *S. aureus* growth dynamics and characterizes a novel high-throughput platform for further exploration of fundamental microwave effects on biological systems.

This article is part of the theme discussion meeting issue ‘Microwave science in sustainable technologies’.

## 1. Introduction

The widespread application of microwaves in biomedical and industrial applications necessitates thorough research into their biological effects. The effect of generating a comprehensive understanding of these effects is twofold: (i) optimization of industrial processes (e.g. sterilization and decontamination in food and healthcare [1,2]), and (ii) improved regulation of safe exposure limits (e.g. informing public health policy).

Research into the non-thermal effects of microwaves has been ongoing since the advent of radar technology during WWII and the use of domestic and industrial microwave heating in the 1950s. Despite this, conclusive evidence of their biological effect is lacking, with many biological studies reporting inconclusive or contradictory findings [3–8]. For example, radar operators reported hearing phenomena, which were later attributed to thermo-acoustic effects [9,10]. While many argue that microwave effects on biological systems are purely thermal [3,4], others present evidence to support a non-thermal mechanism [5–8]. These extend across a range of biological systems. For example, Afaghi *et al*. [11] showed that the spike protein of SARS-CoV-2 virus particles was denatured under non-thermal microwave radiation. In the aforementioned study, non-thermal effects were studied by cooling the samples to counteract electric-field- (*E*-field)-induced heating effects [11]. Conversely, a study conducted by Rougier *et al*. [5] reported non-thermal effects on *Escherichia coli* cell membranes by comparing microwave cavity heating with conventional (water bath) heating. It was concluded that the modifications of cell membrane integrity measured could not be explained by heating alone [5]. A thermocouple was used to measure the bulk temperature of the suspension, meaning that localized heating may have been overlooked. The high degree of dielectric contrast present in biological samples on a microscopic level means that local temperatures could be elevated relative to that measured using conventional thermometry. This means that microscopic temperature probes are needed to rule out localized thermal effects at the cellular scale.

The difficulty in isolating the thermal and non-thermal effects contributes to the controversy and highlights a critical gap in our knowledge. The examples noted above highlight the importance of implementing adequate thermal controls in experiments where microwave-induced heating is encountered [12]. Currently, the International Commission on Non-Ionizing Radiation Protection (ICNIRP) recognizes and implements only the thermal effects of microwaves into their guidelines for safe exposure in the greater than 100 kHz range [13]. This stands at 2 W kg^-1^ for the general public, averaged over 10 g (local head and torso) for 6 min and resulting in 2°C heating. This is a factor of 10 lower than the known threshold for tissue damage. Another major challenge in such studies is ensuring consistent microwave field exposure across samples and replicates. For example, cylindrical cavity resonators (e.g. TM_010_ mode [14]) offer precise *E*-field control but have limited sample capacity unless much lower frequencies are used. Multimode cavities such as domestic microwave ovens accommodate greater sample volumes but suffer from chaotic *E*-field distributions, which reduces the repeatability and reliability of results.

Bacteria offer a useful and accessible model system in which to study the non-thermal effects of microwaves. The bacterium of choice in this study is *Staphylococcus aureus*, known for its clinical relevance as an antimicrobial-resistant pathogen which can cause sepsis. This is one of the best-studied Gram-positive bacteria, with well-defined *in vitro* growth dynamics and extensive genomic and metabolic characterization.

This study documents the design of a microwave waveguide applicator capable of exposing 96 samples (via a 12 by 8 microtitre array) containing *S. aureus* bacteria simultaneously to a quantifiable microwave field at 2.45 GHz. The non-resonant nature of the probe means that aqueous sample volume can be increased, which would otherwise reduce the quality factor of a resonant applicator such as a cavity, which becomes difficult to tune for high sample volumes. Furthermore, the length of the applicator can be increased without altering the applied *E*-field strength, which is uniform along this length. There is a sinusoidal modulation of the *E* field across the width of the waveguide but this is taken into account in our studies. This instrument facilitates the investigation of non-thermal microwave effects, providing a platform for future research that could lead to innovative solutions for biomedical challenges. As well as characterizing the operation of the waveguide, we aimed to measure the non-thermal effects of the microwave *E* field at 2.45 GHz on the growth and metabolic response of *S. aureus*. Ultimately, such data may lead to new control strategies for infections caused by this pathogen.

## 2. Methodology

### (a) TE_10_ waveguide design and fabrication

**Figure 1.**
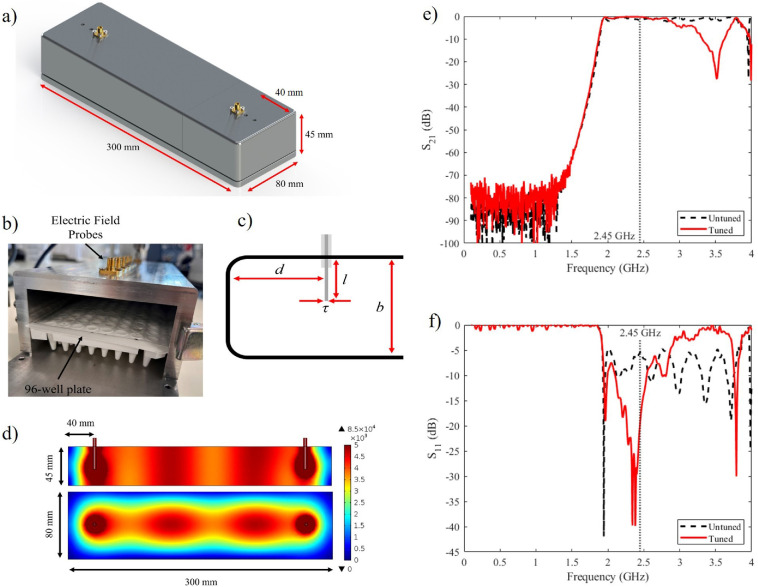
SolidWorks computer aided design image of the rectangular waveguide with internal dimensions: 80 mm width, 45 mm depth and 300 mm length , made from aluminium (a). An image of the open waveguide, showing a 96-well plate inside as well as the *E*-field probes (b). Diagram of the electric probe transition from the coaxial cable into the rectangular waveguide. The dimensions *d*, *l* and *τ* can be varied to optimize the S_11_ and S_21_ of the device (c). COMSOL simulation of the *E* field in a rectangular waveguide. The electric probe transitions cause a variation in the *E* field along the length, quantified by the VSWR (d). An S_21_ plot showing the effect of a tuning screw on the operation of the waveguide from 0 to 4 GHz with a change from −1.6 to −0.4 dB at 2.45 GHz (e). An S_11_ plot showing a change from approximately −6 to −20 dB (f).

A TE_10_ rectangular waveguide was designed with the following dimensions: width 80 mm, depth 45 mm and length 300 mm (figure 1*a*). The upper frequency limit is dictated by the excitation of the next highest operational mode, which in this case is the TE_20_, and the lower frequency limit is set by the cut-off frequency [15]. This results in an operating frequency range of 1.88 < *f* < 3.33 GHz, with 2.45 GHz mid-range. The length of the waveguide was chosen to be 300 mm to allow enough distance between the launcher and the samples for the *E* field to be established. To allow ease of access to samples, the waveguide was designed to separate into three segments, a base plate and two top plates. The two top plates are fixed to the base plate using screws. As the vertical cut between the two top plates is a current-carrying connection, fast release clips were added to ensure good electrical contact. The aluminium used has a thickness of 8 mm, which does not affect the operation of the waveguide, as the internal dimensions dictate its characteristics. It also ensures rigidity, durability and allows for future amendments, such as adding further holes for tuning and probing.

To couple microwave power into and out of the waveguide, an electric probe transition was designed, based on an open-circuit coaxial launcher (figure 1*b*). The theoretical optimum location for the launcher is centred along the width of the waveguide and approximately a quarter of a guide wavelength (*λ*_g_) from the back plate [16] (*λ*_g_ at 2.45 GHz is approx. 190 mm, therefore *λ*_g_/4 is 47.5 mm). The SubMiniature version A (SMA) launcher footprint was positioned at 45 mm from both back plates and a further two holes were drilled to allow for tuning screws. The aim in the transition design is to minimize the *E*-field variation along the length of the sample location, quantified by the voltage standing-wave ratio (VSWR). Theoretically, the voltage transmission coefficient (S_21_) would be uniform (i.e. 0 dB) and flat at frequencies near 2.45 GHz, and the voltage reflection coefficient (S_11_) would be zero (i.e. ideally less than −20 dB). The S_11_ and the S_21_ were optimized by varying the following parameters of the electric probes: length (*l*), diameter (*τ*) and distance from the back plate (*d*) (figure 1*c*).

A finite-element method simulation of the TE_10_ waveguide (using COMSOL Multiphysics) with the electric probe transitions can be seen in figure 1*d*, showing the variation of the *E* field along the length of the waveguide, with the red areas indicating an increased *E* field. COMSOL Multiphysics was used to simulate varying transition parameters to ensure optimum S_21_ and S_11_ at 2.45 GHz. These were found to be *l* = 25 mm, *τ* = 1.75 mm and *d* = 45 mm. A tuning screw was used to further improve the operation of the waveguide which causes S_21_ to change from −1.6 to −0.4 dB at 2.45 GHz (figure 1*e*). S11 showed a corresponding decrease from −6 to −20 dB (figure 1*f*).

Standing waves could not be fully eliminated via device optimization, therefore *E*-field probes were implemented along the length of the waveguide. An approximated VSWR was measured with a voltmeter, connected to a radio frequency detector measuring power through SMA connectors, weakly coupled into the waveguide (figure 1*b*). The weak coupling reduced input-to-probe interference, probe-to-probe interference and power loss. As the detector is operated in its square-law region, the approximated VSWR can be calculated from the square root of the maximum and minimum voltage values, yielding a 25% variation of *E*-field amplitude along the length of the samples.

### (b) Experimental set-up and microwave exposure parameters

The microwave power source used in these experiments referred to as the portable microwave applicator (PMA), was developed and built in-house; it is described more thoroughly in the study by Ahortor *et al*. [17]. It is compact, user-friendly and fully portable, making it ideal for interdisciplinary use. A thorough characterization of the pulse input to the waveguide was undertaken to establish the precise nature of microwave exposure. This characterization utilized a power metre (Agilent U2021XA) to measure the output power and an oscilloscope (Keysight Technologies DSO9254A) to look at the waveform in the time domain, with nanosecond resolution.

The microtitre plate array consisted of 12 by 8 wells (AB0900, length 80 mm, width 127 mm, ThermoFisher Scientific) containing Mueller–Hinton broth (MHB, Oxoid) culture medium. All experiments were performed within a 37.5°C (±0.5°C ) cell culture incubator to ensure a consistent thermal background for control and microwave-exposed samples. A fibre-optic temperature probe (LumaSense Technologies) monitored temperature changes in the centre wells of the 96-well microtitre plate, each containing 200 µL MHB. To define the non-thermal exposure parameters, the PMA was used to deliver 25.3 W r.m.s. at varying on/off cycles (pulses) of 1, 2, 3, 4 and 10 µs ON time, over a period of 100 µs, corresponding to duty cycles of 1, 2, 3, 4 and 10%, respectively. Each duty cycle variable was conducted independently in triplicate, and the temperature was measured over 50 min, allowing sufficient time for the temperature to equilibrate within the microtire wells containing MHB. The pulsing parameters that induced no measurable increase in temperature were selected for non-thermal bacterial investigations.

The *E*-field distribution across the 96-well microtitre plate within the TE_10_ waveguide was evaluated by monitoring the resulting thermal distribution using a thermal imaging camera (Micro-Epsilon TIM 640, 640 × 480 pixels resolution and an accuracy of ±2°C or ±2%, whichever the greater) since we expect the temperature rise to be proportional to the local value of *E*^2^. Each well of the 96-well plate contained 200 µl water, pre-incubated at 4°C. The PMA was used to deliver 25.3 W continuous exposure (i.e. without pulsing) at 2.45 GHz into the waveguide for 10 min. The temperature within the wells of the 96-well plate was measured before and after the microwave exposure.

### (c) Organism and culture

*Staphylococcus aureus* N315 (methicillin-resistant strain isolated in 1982 [18]) was cultured routinely overnight (16 h) in 10 ml MHB at 37.5°C (±0.5°C). Following incubation, each broth was diluted to an absorbance of 0.08−0.1 (0.5 McFarland standard) at 600 nm (Jenway 6305 UV/visible spectrophotometer). Three independent overnight broth cultures were prepared for biological replicates (*n* = 3), while technical replicates were generated from the same overnight broth (*n* ≥ 6).

### (d) Bacterial growth monitoring through optical density measurements

Optical density is a measure of the turbidity of bacterial cultures and is used to infer an estimate of population density. An increase in optical density typically indicates bacterial growth over time. This measurement captures the density of all bacteria (living and/or dead) within the culture medium. Overnight cultures of *S. aureus* were further diluted 1 : 200 in MHB to achieve a starting cell density of approximately 10^5^ CFU mL^−1^. Two hundred microlitre aliquots of the diluted culture were transferred into each well of two 96-well plates and sealed using adhesive plate seals (ThermoFisher Scientific) to prevent sample evaporation and contamination. One plate was placed inside the waveguide, transferred to the 37.5°C (±0.5°C) cell culture incubator and exposed to 2.45 GHz microwave at 25.3 W with varying duty cycles of 0.4, 0.8, 0.9, 1.2 and 1.5%. A second identical plate was set up and placed inside the same incubator, but outside the waveguide. This provided an unexposed thermal control. After the 24 h incubation, the optical densities of each well across both plates were measured at 600 nm (TECAN Infinite 200 Pro Plate Reader). Each well was then independently diluted 1 : 10 000 in MHB and incubated in a bioanalyser (Bioscreen C Reader, MBR) that measured the optical density at 600 nm at 15 min intervals for 24 h at 37°C, with shaking before each reading to ensure homogenization of cells. Each experiment was performed in triplicate to generate three biological replicates. Each biological replicate consisted of six technical replicates. Blank controls of MHB without cells were included as a background measurement and sterility check.

### (e) Viable cell counts

Unlike optical density measurements, viable cell counts provide an estimate of the number of living bacterial cells within the culture. *S. aureus* cell counts were determined using the Miles and Misra method [19]. For this colony, counts were conducted on Mueller–Hinton agar (Oxoid) immediately after 24 h incubation.

### (f) Redox and membrane potential measurements

The fluorescent probes (all ThermoFisher Scientific) employed were 2′,7′-dichlorodihydrofluorescein diacetate (H2-DCFDA), tetramethylrhodamine ethyl ester perchlorate (TMRE) and monochlorobimane (MCB), which measure intracellular reactive oxygen species (ROS), cellular membrane potential and low molecular weight thiols, respectively. Collectively, they provide an overview of the cellular redox state [20–22]. Following a microwave exposure of 25.3 W at the highest chosen duty cycle, 1.5% (1.6 µs ON and a time period of 206 µs), or incubated control plate, 200 µL cells were separately incubated with 0.25 µM of each fluorescent probe in quadruplicate for 30 min at 37°C (±0.5°C). After incubation, cells were pelleted through centrifugation (4000 r.p.m., 10 min), and the first cell-free supernatant was collected for analysis. Cells were washed three times in phosphate-buffered saline (PBS, pH 7.2) through repeated centrifugation; the resulting cell pellet was also analysed. Fluorescence readings from the cell-free supernatant and final cell pellet were collected using the TECAN Infinite 200 Pro Plate Reader at the wavelengths listed in table 1. All assays were repeated in triplicate for biological replicates, each consisting of four technical replicates.

**Table 1.** Excitation (*λ*_ex._) and emission (*λ*_em._) wavelengths for each fluorescent probe.

probe	*λ*_ex._ (nm)	*λ*_em._ (nm)
H2-DCFDA	495	520
TMRE	550	570
MCB	390	490

### (d) Statistical analysis

Statistical analysis was performed using GraphPad Prism software [23] using normally distributed data (determined using the Anderson–Darling normality test). Statistical significance between microwaved and control groups was tested using either a *t*‐test or a two-way ANOVA, depending on the number of independent variables. For each experiment, biological and technical replicates were pooled for statistical analysis.

## 4. Results

### (a) TE_10_ waveguide characterization

Temperature characterization of the 96-well microtitre plate following microwave exposure within the waveguide showed non-uniform temperature distribution across the wells with a 14°C increase in the centre wells compared to the outer wells when plates were pre-incubated at 4°C (electronic supplementary material, figs. S1a–c). Elevated temperature was expected at continuous microwave exposure due to the cross-sectional *E*-field distribution set-up within the TE_10_ rectangular waveguide. At a power level of 25.3 W, the peak *E* field (*E*_0_) = 4.05 kV m^−1^ in the centre of the waveguide; *E*_0_ decreases to approximately half this value in the outer wells. All subsequent biological experiments were pulsed to maintain non-thermal exposure conditions. Electronic supplementary material, fig. S1d shows the position of the three biological replicates across the 96-well plates for reference.

### (b) Microwave pulse characterization

**Figure 2.**
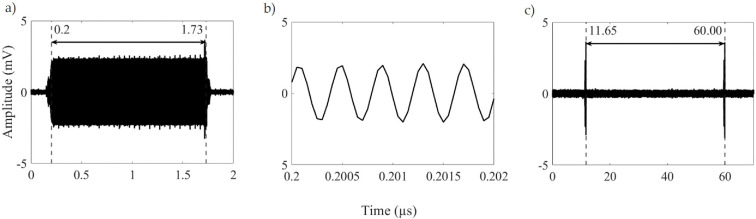
Measured waveform when the PMA is set to 3 µs ON with a 50 µs time period. (a) ON time is shown, including the rise and fall time which are too small to measure on this timescale. The ON time is measured to be approximately 1.53 µs. (b) The same pulse over a much shorter period showing the frequency of the signal is shown and (c) the overall time period which is measured at approximately 48.35 µs.

The PMA was comprehensively assessed to ensure accurate and consistent exposure levels during testing. Analysis of the error between set and measured parameters revealed a consistent offset of 1.4 µs in ON time across a wide range of settings. For instance, when configured for a 3 µs pulse, the measured ON time was 1.6 µs, as shown in figure 2*a*. As a result, exposure settings were adjusted based on the measured ON time. Figure 2*b* illustrates the sinusoidal waveform at 2.45 GHz during the pulse ON interval, confirming oscillating microwave excitation during this phase. Figure 2*c* shows a 3.3% error in the pulse period, with a measured value of 48.35 µs compared to the set value of 50 µs which is in an acceptable range. Optical fibre temperature characterization of microwave-induced heating in the experimental set-up (200 µL MHB) indicated significant heating at 10% duty cycle (electronic supplementary material, fig. S2), defining this as the upper duty cycle limit. Table 2 lists the measured pulse parameters (ON time, period and pulse frequency) across the tested duty cycle range, confirming consistency with non-thermal exposure conditions for subsequent biological experiments. Furthermore, the selected ON times and periods were chosen to examine the potential influence of these individual parameters on bacterial growth, as well as pulse frequency.

**Table 2.** Measured microwave pulsing parameters used in biological experiments.

duty cycle (%)	ON time (µs)	period (µs)	pulse frequency (1 /*T* = kHz)
0.4	0.5	105.7	9.4
0.8	1.6	207.8	4.8
0.9	0.5	51.9	19.2
1.2	2.6	207.8	4.8
1.5	1.6	105.7	9.4

### (c) Microwave exposure increases *S. aureus* optical density, but not viable cell counts

**Figure 3.**
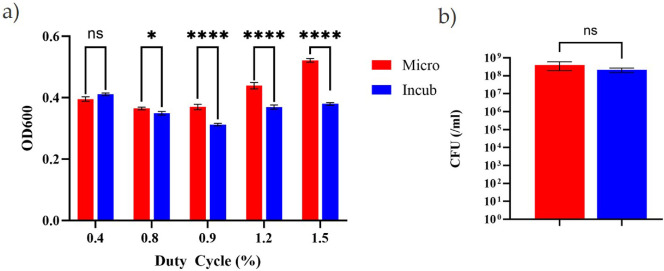
The optical density of *S. aureus* after being incubated at 37°C (blue, control) or microwaved at 2.45 GHz with a power of 25.3 W with a varying duty cycle (red), *n* = 36. The error bars show the s.e. across the 12 samples in each row for each of the three biological replicates. The results of unpaired *t*-tests can be seen above with ’ns’ denoting no significance (*p* > 0.05) and asterisks indicating increasing significance, *p < 0.05, **p < 0.01, ***p < 0.001 and ****p < 0.0001. (a) The number of colony forming units per millilitre (CFU mL^−1^) for incubated samples (blue) and microwaved samples (red) exposed to 1.5% duty cycle (1.6 µs ON with a 105.7 µs period) with error bars representing the standard error across all replicates. Unpaired *t*‐test results show no significance (ns) with a *p*-value of 0.0562, *n* = 18 (b).

Optical density (OD600) measurements from each 200 µL cell suspension within the 96-well microtitre plates immediately after 24 h microwave exposure or incubation at 37°C (±0.5°C) are shown in figure 3*a*. The figure shows no significant difference between the microwaved and control samples at 0.4% duty cycle (0.5 µs ON with a 105.7 µs period). However, significant differences (*p* < 0.05) were observed for exposures at 0.8% duty cycle (1.6 s ON with a 207.8 µs period). Greater significance (*p* < 0.0001) was observed for the remaining duty cycles; 0.9% (0.5 µs ON with a 51.9 µs period), 1.2% (2.6 µs ON with a 207.8 µs period) and 1.5% (1.6 µs ON with a 105.7 µs period), indicating a significantly higher OD600 in the microwaved samples compared to the incubated controls. Bacterial optical density measurements are therefore directly proportional to increasing duty cycle immediately after microwave exposure. In-depth analysis of the OD600 distribution across the microwaved and incubated 96-well plates (electronic supplementary material, figs. S3a–e) revealed that bacterial suspensions with approximately half the *E*-field exposure (outer rows, electronic supplementary material, figs. S1a–d) showed significantly higher OD600 compared to those with the higher exposure (centre rows, electronic supplementary material, figs. S1a–d) and the incubated positive control (PC), as summarized in electronic supplementary material, fig. S3. Furthermore, the difference in OD600 between the inner and outer wells increased linearly with pulse frequency (from 4.8 to 9.4 kHz). Cell counts were also conducted on *S. aureus* to determine the number of viable colony-forming units per millilitre (CFU mL^−1^) in each sample immediately after 24 h microwave exposure or incubation (PC). Figure 3*b* shows no significant difference in CFU mL^−1^ between the microwaved and incubated controls.

### (d) Microwaves alter *S. aureus* growth dynamics post-exposure

**Figure 4.**
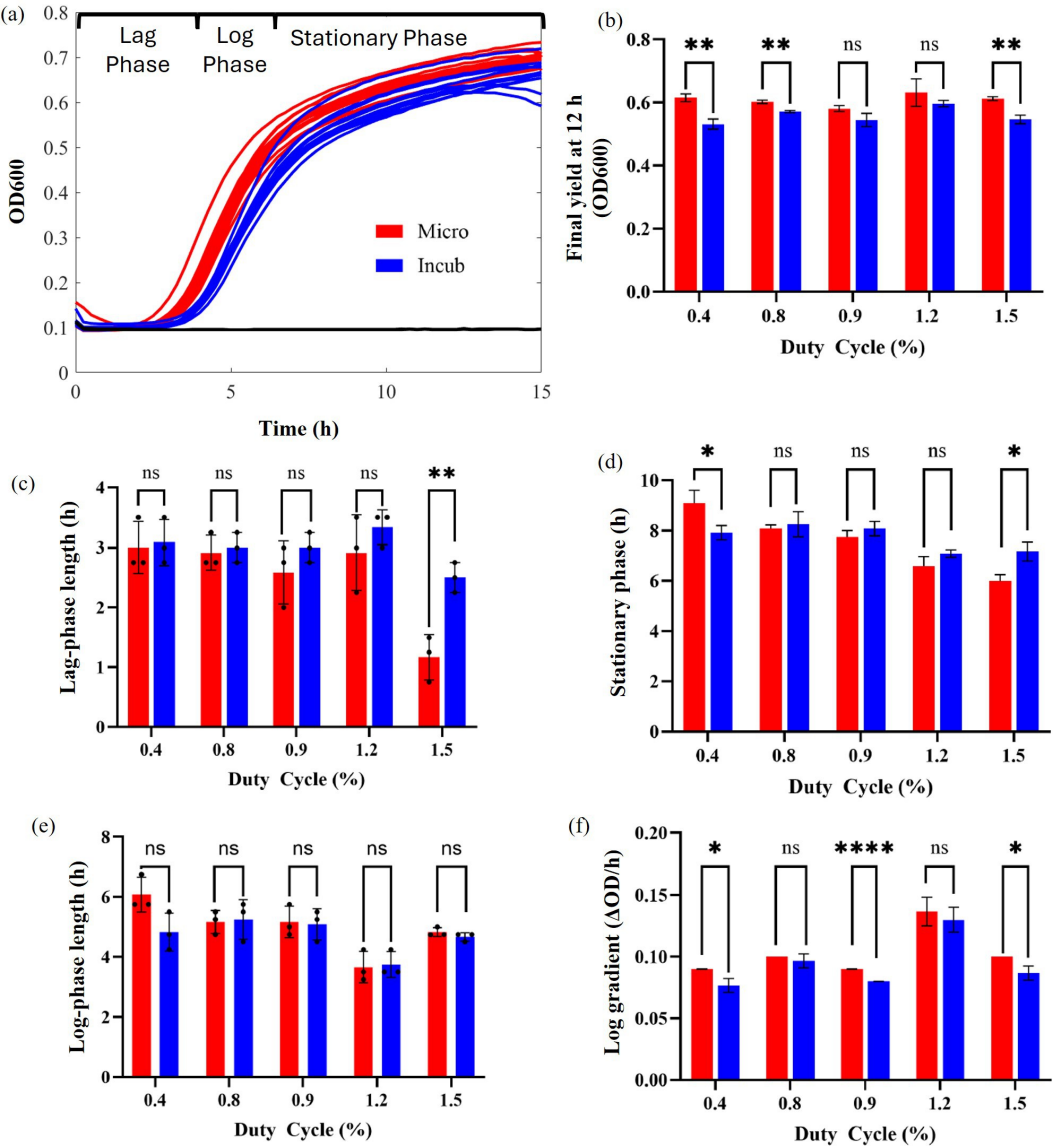
*S. aureus* growth curves after 24 h incubation at 38°C (blue, control) or exposure to 2.45 GHz microwaves (red). The black lines are negative controls which only contain MHB and prove a sterility check, *n* = 90 (a). An in-depth analysis of *S. aureus* growth curves at varying microwave duty cycles compared to incubated controls: final yield (OD600 at 12 h, b), lag length (c), time stationary phase reached (d), log length (e) and log gradient (f). Error bars represent standard deviation, and significance is indicated by *p*-values from multiple unpaired *t*-tests. ’ns’ denotes no significance (*p* > 0.05) and asterisks indicate greater significance, *p < 0.05, **p < 0.01, ***p < 0.001 and ****p < 0.0001, *n* = 36.

All microwaved-exposed *S. aureus* samples exhibited accelerated growth rates and higher optical densities post-exposure compared to the incubated control samples (figure 4). This trend was particularly pronounced for the highest duty cycle tested of 1.5%. The three main phases of bacterial growth are summarized in figure 4*a*. These are the lag phase (a preparatory period where cells are adjusting to their environment), the log phase (a period of exponential growth) and the stationary phase (where the number of dividing cells equals the number of dying cells). An in-depth break down of these phases is shown in figure 4*b*–*f*, for the different duty cycles tested. There is no consistent dose-dependent relationship between increasing duty cycle and each of the growth kinetics analysed. However, a duty cycle of 1.5% led to a significant increase in final yield (OD600 at *T* = 12 h, figure 4*b*) and log gradient (figure 4*f*), and significant decreases in lag-phase length (figure 4*c*) and time taken to reach stationary phase (figure 4*d*). There was no significant difference in log-phase length (figure 4*e*). Generally, the shorter pulse durations (0.5 and 1.6 µs) resulted in more significant changes to growth curve dynamics, particularly for log gradient (figure 4*f*). Log gradient was also directly proportional to pulse frequency (see table 2 for conversions).

### (e) Microwave exposure changes the redox state of *S. aureus*

**Figure 5.**
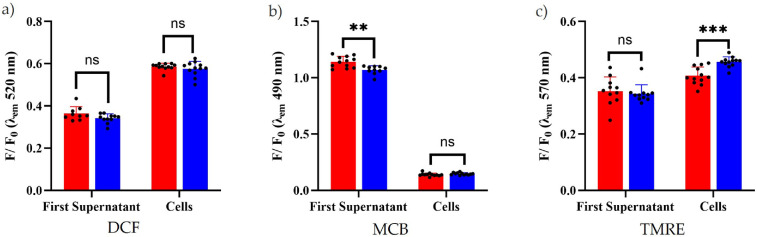
Normalized fluorescence response from both the first supernatant and cells of microwaved and incubated *S. aureus* bacteria, each replicate is represented a black circle. Fluorescence readings were normalized to *F*_0_, the baseline measurement of media and probe alone. Microwaved samples are shown in red and incubated controls are shown in blue for ROS (DCF, a), low molecular weight thiols (MCB, b) and membrane potential (TMRE, c). The results of unpaired t-tests are represented by black lines above the charts, where ns denotes no statistical difference, *p* > 0.05, and asterisks indicate increasing statistical significance, *p* < 0.05, **p < 0.01, ***p < 0.001 and ****p < 0.0001. *n* = 12.

The fluorescent response from the *S. aureus* cell pellets and cell-free supernatants loaded with the fluorescent redox indicators are shown in figure 5. There was no significant difference in H2-DCFDA fluorescence intensity between the microwave and control samples, suggesting no change in cellular (pellet fraction) or extracellular (supernatant) ROS (figure 5*a*). Extracellular MCB fluorescence was significantly higher in the supernatant from the microwaved bacteria than that of the incubated controls (*p* = 0.0012, figure 5*b*). This indicates elevated levels of low molecular weight thiols produced by microwaved cells during exposure, compared to incubated controls. There was no significant difference in intracellular MCB fluorescence between microwaved and incubated cell pellets. There was also no significant difference in extracellular TMRE fluorescence between the microwaved and control supernatants. This was expected as TMRE is a membrane potential indicator and therefore lacks a target in the cell-free extracts. However, the cellular fraction (pellet) exhibited significantly lower fluorescence compared to the PCs (*p* < 0.0001, figure 5*c*).

## 5. Discussion

The primary objective of this study was to design a high-throughput microwave applicator to study the biological effects of microwaves. In characterizing the designed and fabricated TE_10_ waveguide applicator, we demonstrate putative non-thermal effects of pulsed microwave fields on *S. aureus* growth kinetics and redox metabolism.

Microwave pulsing parameters at 2.45 GHz were carefully selected to avoid a measurable temperature increase within the exposed bacterial suspension. Temperature characterization of the 96-well plate after microwave exposure revealed non-uniform *E*-field distribution (electronic supplementary material, fig. S1), where outer wells experienced approximately half the *E*-field intensity as inner wells (peak *E* field = 4.05 kV m^−1^). Temperature differences between the inner and outer wells was eliminated by placing the waveguide within the same cell culture incubator as the PCs for microwave exposure experiments. This therefore provided a uniform background thermal level of 37°C (±0.5°C) across all samples. Furthermore, the transmission coefficient (S21) of this two-port waveguide (figure 1*e*), is approximately equal to 1, indicating that almost all the power applied to the waveguide is exiting to port 2. This means that the degree of microwave absorption in the sample is minimal. This minimal absorption may lead to subtle localized temperature rises within cells, owing to the non-uniformity of the dielectric properties, which cannot be quantified using our set-up. This would require a highly local (and sensitive) temperature measurement. In such instances where the pulsed duty cycle is small, microwave energy is still delivered to the sample, but its finite heat capacity limits the rate of temperature rise.

Subsequently, all non-thermal microwave pulsing parameters tested above 0.4% duty cycle resulted in an increased optical density of *S. aureus* bacteria, when compared to the incubated controls (figure 3*a*), suggesting increased bacterial growth during the 24 h exposure. However, as viable cell counts (CFU mL^−1^) did not differ significantly between microwaved and incubated samples (figure 3*b*), an increase in growth is unlikely to explain this finding. Instead, the increase in optical density observed could be an indication of an increase in cellular debris from dead cells or an increase in cell size. The difference in baseline OD600 values across the inner and outer wells of the 96-well plate at 1.5% duty cycle (electronic supplementary material, fig. S3e) could be indicative of a power threshold, above which microwave exposure becomes stimulatory across the wells. We did not measure any bulk increase in temperature within the wells at this higher duty cycle; however, we cannot rule out the presence of localized heating which may account for this result. Interestingly, the OD600 in the outer wells, with approximately half the *E*-field exposure, were significantly higher than those within the inner wells, the latter displaying similar OD600 values to the incubated controls (PC, electronic supplementary material, figs. S3a–d). Such differences were not observed for the PCs (electronic supplementary material, fig. S3f), thus ruling out the effect of factors associated with well location on the plate (e.g. sample evaporation in the outer wells). The difference in OD600 between the inner and outer wells for microwaved samples displays a linear relationship with increasing pulse frequency from 4.8 to 19.2 kHz. There was no obvious association between pulse ON time and period. Whilst the duty cycle relates to the average power exposure of the sample over time, the pulse frequency reflects the number of microwave pulses per second. It is therefore possible that two distinct microwave-induced effects are evident: [1] a power-dependent effect resulting from the imposed duty cycle, possibly caused by localized temperature increases at the highest duty cycle (i.e. 1.5%), and [2] a pulse frequency-dependent effect which is non-thermal as it is inversely proportional to *E*-field strength and hence temperature.

*S. aureus* growth-curve analysis post-microwave exposure revealed accelerated growth rates in the microwaved samples, which indicates that microwaves have a sustained effect on bacterial growth, even after they are no longer applied (figure 4). The highest duty cycle tested of 1.5% consistently showed a change in growth curve parameters resulting in more rapid growth kinetics (figure 4). The increased growth kinetics observed for the log gradient (figure 4*b*) also increased linearly with pulse frequency. Therefore, both the power (duty cycle) and pulse frequency-dependency observed immediately after microwave seem to have a prolonged effect on *S. aureus* growth kinetics up to 12 h after exposure. The effect of plate position (and therefore *E*-field intensity), however, did not significantly alter bacterial growth curves, despite the measured differences in OD600 immediately after exposure. The optimal growth range of *S. aureus* is between 30 and 37°C. Prolonged incubation above 42°C is not recommended. Electronic supplementary material, fig. S4 shows that small changes in incubation temperature (less than 2°C) do not significantly affect *S. aureus* optical density and growth kinetics as is case for the microwave experiments described.

While many studies report that microwave exposure results in reduced bacterial growth rates or even bacterial inactivation [24,25] a small number of studies align with the findings of this research. For instance, Amanat *et al*. [26] demonstrated that *Lactobacillus acidophilus* and *L. casei,* which lik*e S. aureus* possess a Gram-positive cell wall structure, showed significantly increased proliferation and lactic acid production when exposed to 2.4 GHz electromagnetic radiation from a commercial Wi-Fi router for up to 1 h. Similarly, Cohen *et al*. [27] found that exposing Gram-negative *Escherichia coli* bacteria to 99 GHz microwaves for 19 h resulted in a nearly half-order-of-magnitude increase in colony-forming units compared to controls. In terms of microwave pulse parameters, extremely rapid nanosecond pulsing at high *E*-field intensities have been shown to inactivate *S. aureus* and *E. coli* cells through cell surface damage, increasing ROS and decreasing thiol levels [22]. The increased growth kinetics observed for the longer microsecond pulse durations employed in the current study emphasizes the importance of in-depth analysis of a range of microwave parameters on biological systems for biomedical applications.

Fluorescence assays provided initial insights into the underlying cellular mechanisms causing the increased growth kinetics observed. The membrane potential indicator TMRE revealed a decreased signal indicating a reduction of membrane potential in microwaved cells. This could be indicative of a range of biological phenomena including lower metabolic activity or cellular stress. Stratford *et al*. [28] showed that exogenous direct current (DC) *E*-field stimulation of bacteria led to differential bacterial membrane responses. Actively proliferating cells underwent hyperpolarization, whereas inhibited cells displayed a relaxation response. In the current study, the cells assayed had been in culture for 24 h and therefore had reached stationary phase growth. Moreover, microwaved cells displayed increased growth kinetics, therefore the decreased TMRE signal (and therefore membrane potential), could be indicative of this inhibitory growth phase, which is accelerated by microwave exposure.

Kubo *et al*. [29] discuss the non-thermal effects of *E* fields on microbial inactivation, focusing on electroporation of cell membranes. When an external *E* field at DC voltage is applied, pores can form in the membrane if the *E*-field magnitude exceeds a critical value (approx. 500 kV m^−1^), dielectric breakdown occurs, leading to irreversible cell damage. However, moderate *E*-field amplitudes similar to the levels used in the current study, are known to cause reversible permeabilization, temporarily disrupting the cell membranes while maintaining cell viability [17]. This may affect microbial growth and metabolic activity. Membrane potential regulates a wide range of bacterial physiology including cell division [30] and modulates the distribution of several cell division proteins [31], which could link the reduced membrane potential observed to the higher growth rate observed in this study.

The MCB assay showed increased levels of low molecular weight thiols in the cell-free extracts of microwaved cells (figure 5*b*). Low molecular weight thiols play a key role in maintaining redox homeostasis in *S. aureus,* implying an elevated stress response during microwave exposure. Hartl *et al*. [32] investigated the role of glutathione (GSH) in cellular processes, particularly cell division, using a mutant strain of *Caulobacter crescentus* with reduced GSH production. They observed that the mutant cells were smaller than the control strain, otherwise known as the wildtype, and exhibited disrupted coordination between cell growth and division, as evidenced by optical density growth curves. These findings suggest that the increased thiol levels observed in this study may explain the higher optical density readings observed without a corresponding increase in viable cell count. This could therefore imply an increase in cell size rather than number. In addition, the observed increase in thiols could explain the enhanced growth rate observed under these conditions. Although no direct increase in ROS was measured using the H2DCF-DA probe, thiols are known to protect the cell membrane from oxidative stress [33], therefore the increase in thiols may be a compensatory response to the reduced membrane potential observed rather than ROS production directly. This suggests that elevated thiol levels could be a protective mechanism to maintain membrane integrity during microwave exposure. Collectively, the fluorescence assay results suggest that microwaves reduce cellular membrane potential and increase low molecular weight thiol production by *S. aureus*, which indicates an overall change in cellular redox state, accompanying the accelerated growth kinetics observed.

Finally, while all efforts were made to omit the thermal effects of microwave exposures in our experimental design, we were limited in our ability to measure heating at the localized (i.e. cellular) scale. Our experiments relied on bulk temperature measurements generated through the optical fibre thermal probe and thermal imaging. These allowed real-time monitoring of heating and greater spatial resolution post-microwaving, respectively. Conducting all experiments within the same cell culture incubator provided the same thermal background for both microwave and control groups (i.e. 37°C ±0.5°C). This, coupled with the fact that no heating was measured for any of the microwave parameters employed, provides high confidence of no bulk heating effects during our experiments. Localized heating at the cellular scale, for example using temperature-sensitive fluorescence probes and micro-Raman, should be explored in future studies to verify the apparent non-thermal effect described here.

## 6. Conclusions

A TE_10_ waveguide microwave applicator was designed, fabricated and optimized for high-throughput investigation into the non-thermal effects of microwaves on bacterial growth. The waveguide utilized the symmetry of the *E*-field distribution of a rectangular waveguide to allow quantifiable exposure of *S. aureus* cultures within a 96-well plate to microwave *E* field. Transitions were designed to allow coupling of microwave power in and out of the waveguide and tuning screws were used to reduce the VSWR.

This research contributes to the growing body of knowledge on the non-thermal effects of microwaves and provides novel tools to study these effects. The study demonstrates that microwave exposure at microsecond pulsing parameters significantly affects the growth and redox dynamics of *S. aureus* bacteria. These findings persist up to 12 h after the microwaves are removed, or approximately 36 generations (assuming a doubling time of 20 min). This represents a persistent non-thermal effect, which with further characterization of the underlying mechanism, could be fine-tuned to a range of biomedical and industrial applications, from disinfection to optimization of fermentation systems and detection.

## Data Availability

Data will be made available upon request to the corresponding author. Supplementary material is available online at [34].
